# Developing an *in vitro* osteochondral micro-physiological system for modeling cartilage-bone crosstalk in arthritis

**DOI:** 10.3389/fimmu.2025.1495613

**Published:** 2025-05-26

**Authors:** Kyra W. Y. Smith, Stephanie L. Fung, Hsin-Fang Wu, Irene Chiesa, Giovanni Vozzi, Carmelo De Maria, Riccardo Gottardi

**Affiliations:** ^1^ Department of Bioengineering, School of Engineering and Applied Science, University of Pennsylvania, Philadelphia, PA, United States; ^2^ Pulmonary and Sleep Medicine, Children’s Hospital of Philadelphia, Philadelphia, PA, United States; ^3^ Department of Information Engineering and Research Center E. Piaggio, University of Pisa, Pisa, Italy; ^4^ Division of Otolaryngology, Department of Surgery, Children's Hospital of Philadelphia, Philadelphia, PA, United States; ^5^ Department of Otorhinolaryngology, Head and Neck Surgery, Perelman School of Medicine, University of Pennsylvania, Philadelphia, PA, United States; ^6^ Department of Orthopaedic Surgery, Perelman School of Medicine at the University of Pennsylvania, Philadelphia, PA, United States; ^7^ Ri.MED Foundation, Palermo, PA, Italy

**Keywords:** arthritis, *in vitro* models, tissue engineering, crosstalk, organ on a chip, disease modeling, cartilage, inflammation

## Abstract

**Introduction:**

Arthritis, a disease affecting over 50 million adults in the United States, encompasses many different conditions involving joints and surrounding tissues. Disease development, progression, and subsequent treatment is dependent on many different factors, including the relationship between adjacent tissues and the immunological signals involved. A major contributor to disease regulation is the crosstalk between the cartilage and the bone in joints, as well as their reaction to immune factors such as cytokine signaling and macrophage mediation. Studying cartilage-bone crosstalk in arthritis development can be difficult, as controlling immunological factors *in vivo* is challenging, but *in vitro* models often lack multi-tissue relevancy.

**Methods:**

To fix this, we developed an *in vitro* micro-physiological system using a biphasic bioreactor that supports modeling of multiple tissues. We generated cartilage and vascularized-bone analogs and combined them in the bioreactor to allow diffusion and signaling between them. Using this system, we directly induced inflammation in the cartilage region and studied how crosstalk between the two adjacent tissues contributed to disease progression.

**Results:**

We showed that conditioned media from pro-inflammatory macrophages generated a different inflammatory profile than a simple inflammatory cytokine cocktail. We also showed that the vascularized-bone region became inflamed in response to the cartilage inflammation, verifying crosstalk in the system and successfully modeling the relationship between cartilage and bone in an arthritic environment.

**Discussion:**

This model can be used to further probe the crosstalk between bone and cartilage in arthritis, allowing researchers to tease out the effect of specific inflammatory agents or therapeutics *in vitro*.

## 1 Introduction

Arthritis is a complex disease group that affects bone, cartilage, and the surrounding tissues in articular joints (1, 2). Arthritis is a major cause of disability and afflicts over 50M adults in the United States (3). There are currently few successful therapies for arthritis, most of which are palliative in nature such as physical therapy and non-steroidal anti-inflammatory drugs (4). There has been some progress with disease-modifying drugs that can prevent continued progression of arthritis, especially thanks to the use of monoclonal antibodies, however, none can completely reverse the effects (5). Therefore, continued study of disease progression and therapeutic efficacy is critical.

To design better solutions for the treatment of arthritis, it is important to acknowledge that an articular joint is a complex organ consisting of multiple tissues such as cartilage, bone, vasculature, and synovium (6). Articular cartilage is responsible for dispersing and distributing the load on the joint (7). The primary cells in cartilage are chondrocytes, which are sparsely distributed in a dense extracellular matrix (ECM) whose main characteristic components are collagen type II, glycosaminoglycans (GAGs), and aggrecan (1). Below cartilage and in stark contrast is the subchondral bone, innervated and vascularized, rich in minerals and collagen type I, and populated with osteocytes, osteoblasts, and osteoclasts (8). Bone also encapsulates the bone marrow cavity, which is rich in nutrients and stem cells (9, 10). The synovium, lining the articular joint capsule, contains macrophage-like cells and fibroblast-like cells that clear debris and secrete ECM components and cytokines (11, 12). In a healthy joint, the cartilage, bone, vasculature, and the joint space components undergo constant crosstalk to function as an articular unit. Nutrients from the joint space help support cartilage health and function. The vascularization drives bone remodeling (13), and in turn the bone supports cartilage structure and function (14). These processes can be compromised in arthritis, thus dysfunction in one tissue can negatively affect any of the others. This crosstalk plays an important role in the development and progression of arthritis in articular joints. Inflammatory cytokines in the joint space affect the phenotype of the resident cartilage and bone cells and propagate the disease (11, 15, 16). The cartilage responds to inflammatory cues with the degradation and progressive loss of critical ECM components such as GAGs and collagen type II (16–19). As a result, the subchondral bone experiences changes in mineralization and turnover, and local lesions appear (20–23). Functionally, the cartilage loses its mechanical integrity, and its erosion leads to bone-on-bone contact, which is associated with significant pain for patients. It is clear that when studying arthritic disorders or developing treatments, it is critical to consider tissue crosstalk in response to inflammatory signals and how they may affect each other as well as the ultimate clinical outcomes.

Given the complexity of the interactions in arthritic articular joints, animal models are commonly used to capture the full breadth of physiological functions (24–26). However, animal models have varying degrees of biological similarity compared to humans in both joint structure and immunological profile (27, 28). An example of differing joint mechanics is that human knees can reach full extension, whereas neither cow, sheep, goat, pig, dog, or rabbit knees can even surpass 20° extension (28). Immunologically, there are many differences in immune cell populations and responses between common animal models and humans. Specific examples relevant to this work include the toll-like receptor (TLR) pathway; Seok et al. found that in response to inflammation, activation of genes within the TLR pathway of a mouse model could not accurately predict the activation of genes in humans (29). Notably, mouse monocytes have been shown to lack a TLR4 response to lipopolysaccharide (LPS) (30). More broadly, Vijayan et al. found that mouse macrophages favored oxidative phosphorylation after LPS activation, whereas human macrophages reprogrammed to increase glycolysis (31). Another relevant example is that mouse endothelial cells have been shown to express P-selectin in response to tumor necrosis factor (TNF) and LPS, whereas human endothelial cells were nonresponsive (32). Considering the differences in immune response between human and animal models, it is not surprising that many therapeutics that are successfully tested in animals only have limited success in the clinic (5, 33). For example, mouse and rat studies of a disintegrin and metallopeptidase with thrombospondin (ADAMTS) type 5 inhibitor showed successful prevention of cartilage loss (34), but there was no statistically significant improvement in a human clinical trial (35). In fact, even a Food and Drug Administration (FDA) approved drug, anakinra, only shows modest efficacy in the clinic but poses a risk of infection (36, 37). Similarly, FDA-approved infliximab for TNF inhibition only has about a 50% success rate in clinic, and the response is subtle (38). Hence, while *in vitro* models lose in complexity compared to animals, they offer the unique possibility of using human cell sources, thus gaining biological similarity in that respect. Organ-on-a-chip and *in vitro* models have been advocated for as complementary tools alongside animal models. Additionally, animal models may be costly or inaccessible to many researchers around the globe, and there is increasing drive to reduce or replace the use of animals in scientific research (39, 40).

Given the shortcomings of animal models discussed above, there has been a growing body of research on the use of *in vitro* models to study arthritis progression. However, many of the currently available *in vitro* models have yet to recapitulate the complexity of the multiple tissues in articular joints. It is a challenge to co-culture many different cell types in different matrices, perfuse them with different media to match each cell’s needs, and target the delivery of inflammatory agents. As mentioned earlier, there are important biochemical cues from the synovial fluid that lead to disease progression in cartilage, as well as between the cartilage and the subchondral bone (16, 41, 42). The inflammatory agents in the synovial fluid play a role in arthritis development (12), and the constant signaling between the synovium, cartilage, and bone is thought to sustain disease progression (43). Thus, a more veritable *in vitro* model should be able to capture the crosstalk between bone and cartilage in order to accurately depict disease progression. One approach has been using transwell plates to co-culture combinations of mesenchymal stem cells (MSCs), chondrocytes, and synoviocytes (44), and study the effect that each cell type has on the others in a healthy or diseased environment. However, transwells do not prevent media mixing and cells cultured in monolayers do not capture the three-dimensional (3D) structure of the joint tissues (45). Human cartilage is over 2mm thick, and subchondral bone is similar if not thicker. Diffusion of growth factors and cytokines across such relatively long distances compared to the scale of a cell, and through dense matrices critical to tissue function, is not frequently modeled *in vitro*. To account for this, Samavedi et al. adapted co-culture models in transwells to accommodate a hydrogel with encapsulated macrophages in one compartment and chondrocytes in a hydrogel in the other transwell compartment, to study crosstalk between the two cell types in a more biomimetic, 3D environment (46). The shortcoming of this model is that the two constructs do not interface, so the diffusion of signaling factors occurs through the common medium, whereas *in vivo* the signaling factors diffuse more directly through the tissue (47, 48). To account for direct tissue-tissue contact, biphasic constructs have successfully been created to model the cartilage-bone interface, however the whole construct was still cultured in a single common medium. This does not account for the specific cues each tissue needs or is exposed to *in vivo*, nor does it eliminate the confounding crosstalk factor of signaling diffusion through the medium rather than across the cartilage-bone interface (49, 50). So, while great progress has been made to model arthritis of the articular joint *in vitro*, there is much room for improvement.

In arthritis, the joint is often inflamed, and synovium inflammation, possibly associated with macrophage polarization to a pro-inflammatory phenotype, is thought to be a major driver of arthritic development. The synovial fluid directly interfaces with cartilage; however, few *in vitro* models can separately deliver inflammatory cues to either cartilage or bone. In this work, we adopted one of few approaches leveraging our previously described biphasic bioreactor that allows us to perfuse two different types of media through a biphasic construct without mixing (51, 52). Therefore, we perfused the cartilaginous region of a construct with chondrogenic medium, and the osseous region with osteogenic medium, separately. For our *in vitro* osteochondral construct, we generated cartilage and vascularized bone analogs as described in our previous work (41, 42). The constructs differentiated in the bioreactor independently while maintaining contact to allow crosstalk between the cartilaginous and vascularized osseous regions. Furthermore, the primary key benefit of the bioreactor is the possibility to perfuse the cartilage region only with inflammatory cues modeling an inflamed synovial fluid. We then probed the effects of the inflammatory agents on the cartilaginous layer, and the subsequent response of the vascularized osseous layer to an inflamed cartilage analog. While delivering synovial fluid in our bioreactor is beyond the scope of the study and not feasible due to limited access to samples, we perfused the cartilage region with inflammatory cytokines that are commonly found in synovial fluid of arthritic patients (11). Furthermore, rather than just delivering one or more inflammatory cytokines to the constructs, to better mimic some of the complexity of *in vivo* inflammation, we also tested the effect of macrophage conditioned medium (MCM) to represent the inflammatory signals secreted by synovial-resident macrophages during arthritis (15, 16). Thus, we compared the response to a combination of cytokines that are highly implicated in arthritis with the response to a pro-inflammatory macrophage conditioned medium that represents a complex biological cocktail more similar to synovial fluid (53, 54). We aimed to validate crosstalk in the proposed system and to determine how to best mimic *in vivo* inflammation. We hypothesized that MCM will evoke an inflammatory profile similar but not identical to that of the cytokine cocktail, and that there will be detectable responses in the vascularized-bone region after pro-inflammatory activation of the cartilage region.

## 2 Materials and methods

Reagents were purchased from Thermo Fisher Scientific (Waltham, MA) unless otherwise specified.

### 2.1 Bioreactor fabrication

The bioreactor was fabricated according to Ianetti et al. and Nichols et al. Briefly, the bioreactor was designed using SolidWorks (Waltham, MA) computer-aided design modeling software. The bioreactor was 3D printed using a stereolithography apparatus from 3Dsystems Viper si2 (Rock Hill, SC) with Somos WaterShed XC 111222 (Elgin, IL) resin (51, 52, 55).

### 2.2 Cell culture

Bone marrow derived human MSCs (BM-hMSC) were purchased from Rooster Bio (Frederick, MD) at passage 2 and cultured until passage 5 in Rooster-Nourish expansion media (Rooster Bio, Frederick, MD). Three different donor BM-hMSCs were pooled to create each biological replicate, with a total of three biological replicates total (total number of pooled donors n=9, see **Table 1**). BM-hMSCs were seeded into experimental constructs at passages 5 to 7.

**Table 1:** Age and sex information of bone marrow-derived human mesenchymal stem cells.

	Donor Lot #	Age	Sex
**Pool 1**	227/228	29	F
	310281	26	M
	238/310334	22	M
**Pool 2**	310268	19	M
	310278	29	M
	180	23	M
**Pool 3**	114/310264	20	F
	198/257/310318	23	M
	174/310272	25	M

Human umbilical vein endothelial cells (HUVECS) were purchased from Angio-Proteomie (Boston, MA) and expanded in monolayer on a 0.2% gelatin coating in endothelial growth medium 2 (EGM-2, Angio-Proteomie, Boston, MA). HUVECs were seeded into osteochondral constructs at passage 5.

### 2.3 Construct fabrication

#### 2.3.1 Cartilage construct

Methacrylated gelatin (GelMA) (PhotoGel^®^ ~95% DOM, Advanced Biomatrix, Carlsbad, CA) and Methacrylated hyaluronic acid (MeHA) (PhotoHA^®^ Stiff, Advanced Biomatrix, Carlsbad, CA) were reconstituted to 10% weight/volume (w/v) and 5% w/v respectively, in sterile phosphate buffered saline (PBS) containing 0.15% w/v lithium phenyl-2,4,6-trimethylbenzoylphosphinate (LAP) (Millipore Sigma, Burlington, MA) overnight in a 37°C shaker. Reconstituted PhotoGel^®^ and PhotoHA^®^ were combined 1:1 to create a final solution containing 5% w/v GelMA, 2.5% w/v MeHA, and 0.15% LAP photo-initiator.

BM-hMSCs were suspended in the GelMA/MeHA/LAP solution at 15 million cells/mL, pipetted into ⌀4mm x 2mm cylindrical silicone molds, and photo-crosslinked using a near-ultraviolet (UV) flashlight (395nm, 8 mW/cm^2^) for 1 minute each from the top and bottom. Constructs were cultured in Dulbecco’s Modified Eagle Medium (DMEM) containing 10% volume/volume (v/v) fetal bovine serum (FBS) (VWR, Radnor, PA) and 2% v/v Antibiotic-Antimycotic (Anti-Anti) for 24 hours then changed to chondrogenic medium containing DMEM, 2% v/v Anti-Anti, 10μg/mL insulin-transferrin-selenium (ITS), 40μg/mL L-proline (Millipore Sigma, Burlington, MA), 50μg/mL L-ascorbic acid 2-phosphate (Millipore Sigma, Burlington, MA), and 10ng/mL transforming growth factor-β3 (TGF-β3). The cartilage constructs were differentiated in the plate on a rotating shaker for 14 days prior to the next step, where they either continued differentiation for 14 more days on the plate or were combined with the vascularized bone construct to generate a complete osteochondral construct.

#### 2.3.2 Vascularized bone construct

The bone scaffold was fabricated as described in our previous work (41, 42). Briefly, biomaterial ink containing 10% w/v type A porcine gelatin (Millipore Sigma, Burlington, MA), 50% w/v nanohydroxyapatite (Nano-HAp) (Fluidinova, Maia, Portugal), and 0.2% w/v genipin (Challenge Bioproduct Co., Yun-Lin Hsien, Taiwan) in PBS was 3D printed using a piston-driven extruder 3D bioprinter with sacrificial support material Pluronic acid. 100K BM-hMSCs were seeded onto each bone scaffold and cultured in Rooster-Nourish expansion medium for 7 days. The medium was then replaced with osteogenic medium containing DMEM, 10% v/v FBS, 2% v/v Anti-Anti, 10mM β-glycerophosphate, 50μg/mL L-ascorbic acid 2-phosphate, and 10nM 1α, 25-Dihydroxyvitamin D3 (Enzo Biochem, Farmingdale, NY) for 14 days (**Supplementary Figure 1**). To vascularize the bone construct, HUVECs and BM-hMSCs (120K and 30K per scaffold, respectively) were seeded in the pores of the bone scaffold in a hydrogel composed of 5% w/v GelMA, 0.075% w/v LAP, and diluted Tisseel fibrin sealant (0.0025% w/v fibrinogen, 0.00004% w/v thrombin suspension) (Baxter Inc., Deerfield, IL) in PBS. Hydrogels were thermally crosslinked then photo-crosslinked for 2 minutes each.

#### 2.3.3 Osteochondral construct

**Figure 1:**
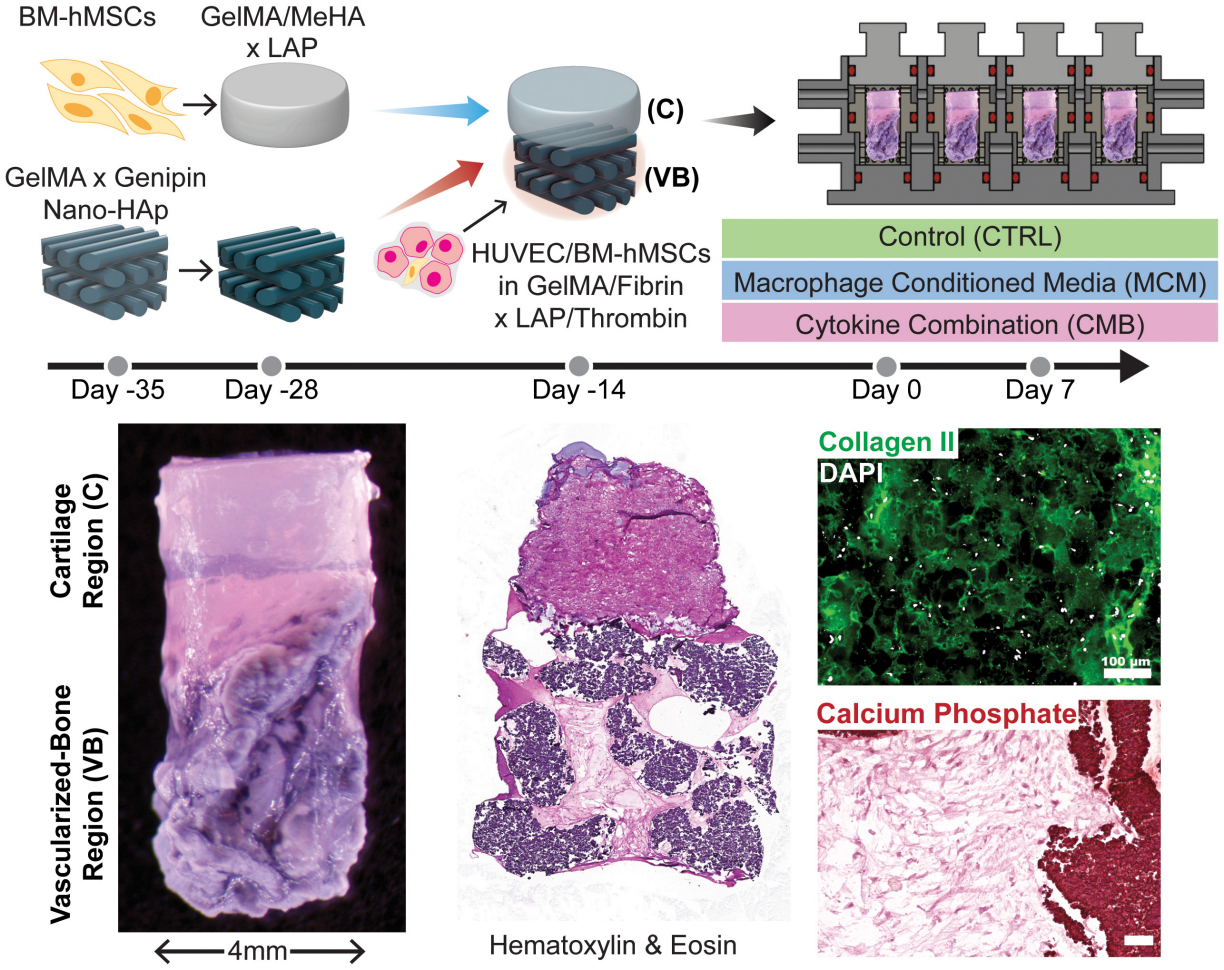
Graphical abstract. Diagram depicting the timeline of setting up the experiment, the experimental conditions, and images showing the construct, with staining for general structure, collagen II in the cartilage region, and calcium phosphate in the osseous region (Scale = 100μm). CTRL medium contains DMEM, 2% v/v Anti-Anti, 10μg/mL ITS, 40μg/mL L-proline, 50μg/mL L-ascorbic acid 2-phosphate, and 10ng/mL TGF-β3. CMB medium contains CTRL medium + 20 ng/mL IL-1β, 100 ng/mL IL-6, and 1000 ng/mL TNF-α. MCM contains CTRL medium + 20x concentrated macrophage conditioned medium (described in 2.4.2).

Complete osteochondral constructs were fabricated after 14 days of pre-differentiation of both the cartilage and the bone analogs. The cartilage analog was placed on top of the vascularized bone construct prior to cross-linking of the fibrin/GelMA with the HUVECs/BM-hMSCs. The construct was thermally crosslinked at room temperature for 2 minutes, then photocrosslinked using a near-UV flashlight for 1 minute each from the top and bottom. The osteochondral construct was then loaded into the biphasic bioreactor and differentiated for another 14 days with chondrogenic medium perfusing the upper chamber and 1:1 osteogenic and vasculogenic media (EGM-2) perfusing the lower chamber (**Figure 1**).

### 2.4 Experimental setup

#### 2.4.1 Bioreactor setup

The bioreactor was connected via silicone tubing (5054K304, McMaster-Carr, Elmhurst, IL) and polypropylene male luer locks (51525K142, McMaster-Carr, Elmhurst, IL) to two 20mL syringes on one side, and two medium perfusion bags (Kiyatec, Greenville, SC) on the other. The system was loaded onto a KDS 220/220P Legacy Syringe Pump (KD Scientific, Holliston, MA) in a cell culture incubator. The medium perfusion bags were filled with 20mLs of the desired medium and the syringe pump was set to withdraw continuously at a rate of 1.2μL/min.

#### 2.4.2 Macrophage conditioned media

Tohoku Hospital Pediatrics-1 (THP-1) monocytes (ATCC, Manassas, VA) were differentiated into macrophages in Roswell Park Memorial Institute (RPMI) medium containing 10% v/v FBS, 1% v/v Anti-Anti, and 100 nM phorbal-12-myristate 13-acetate. Cells adhered in 48 hours. The medium was replaced with complete RPMI-1640 (10% FBS, 1% PSF) for 24 hours. Medium was then changed to polarization medium (fluorobrite-DMEM, 1 mM sodium pyruvate, Minimum Essential Medium Non-Essential Amino Acids, 45mg/L Glutamax, 20 ng/mL interferon gamma (IFN-γ), 100 ng/mL LPS), a highly validated protocol for polarizing macrophages (56, 57). Cells were polarized for 72 hours. Macrophage polarization was confirmed by real time quantitative polymerase chain reaction (RT-qPCR) for M1 genes cluster of differentiation 80 (CD80) (58) and C-C chemokine receptor type 7 (CCR7) (59), and M2 genes C-C chemokine ligand 18 (CCL18) (60) and C-C chemokine ligand 22 (CCL22/MDC) (61) (**Supplementary Figure 2A**). Conditioned medium was collected, pelleted at 500xg to remove cell debris, and the supernatant was transferred to a new tube. Enzyme-linked immunosorbent assay (ELISA) kits for cytokines interleukin-1β (IL-1β), interleukin-6 (IL-6), and tumor necrosis factor-α (TNF-α) were performed according to the manufacturer’s instructions (Peprotech, Cranbury, NJ, **Supplementary Figure 2B**). In each experiment, three dilutions (1:10, 1:100, 1:1000) of the conditioned medium were analyzed by ELISAs. The dilution within the standard curve was chosen for downstream calculations. The conditioned medium was then concentrated 20X using protein concentration columns (3K molecular weight cutoff) per the manufacturer’s instructions. ELISAs were repeated to confirm accurate concentration and endotoxin detection assays were performed to ensure removal of LPS and IFN-γ.

#### 2.4.3 Inflammatory conditions

After 14 days of continued biphasic differentiation in the bioreactor, inflammation was initiated by perfusing the cartilaginous layer with either control medium (CTRL) (chondrogenic medium), MCM (chondrogenic medium with concentrated pro-inflammatory polarized macrophage conditioned medium diluted 20X), or a combination of inflammatory cytokines (CMB) (chondrogenic media with 20 ng/mL IL-1β, 100 ng/mL IL-6, and 1000 ng/mL TNF-α) (STEMCELL Technologies, Cambridge, MA). The lower chamber remained perfused with 1:1 osteogenic and vasculogenic medium. Inflammatory medium was perfused for 7 days with medium refreshed on day 3 and 5. Similarly for cartilage only experiments, the chondrogenic medium was switched out to inflammatory conditions, with medium changed on days 3 and 5.

### 2.5 Histology and immunofluorescence

Samples were fixed in 10% w/v formalin, and cryo-embedded after stepwise washes in 10%-30% w/v sucrose solution. 8μm sections were then re-fixed with 4% w/v paraformaldehyde prior to staining. Hematoxylin and Eosin (Epredia, Kalamazoo, MI) staining was performed to visualize overall structure. Collagen Type II immunofluorescence was performed using rabbit Anti-Collagen II (ab34712, Abcam, Cambridge, UK) and goat anti-rabbit IgG Alexa Fluor™ 555 per the manufacturer’s instructions. For all immunostaining, antigen retrieval was performed with proteinase K for 1 hour at RT, followed by 0.15U/mL chondroitinase (Millipore Sigma, Burlington, MA) and 0.75mg/mL hyaluronidase for 30 minutes at RT. Slides were imaged using a Keyence BZ-X800 (Itasca, IL) microscope with a Nikon (Tokyo, Japan) camera.

### 2.6 RT-qPCR

Samples were flash-frozen and stored dry at -80°C until use. For total ribonucleic acid (RNA) extraction, samples were homogenized in TriZol using a microhomogenizer, then purified using the RNeasy Plus mini kit (Qiagen, Hilden, Germany). Reverse transcription was performed using the SuperScript IV kit. RT-qPCR was performed on a QuantStudio 7 with SYBR Green Master Mix. Primer pairs (Integrated DNA Technologies, Coralville, IA) are reported in **Table 2**. PCR data was reported as -ΔΔCT, normalized to housekeeping gene (HKG) ribosomal protein L13a (*RPL13a*) and pool/donor matched day 0 untreated timepoint. Data is not normalized to the CTRL condition, which is a day 7 untreated timepoint, as some CTRL samples continue to differentiate during 7 days of treatment and this trend is important to show.

**Table 2:** Primer sequences used in RT-qPCR.

Gene	Forward Sequence 5’-3’	Reverse Sequence 5’-3’
**YWHAZ**	ACCGTTACTTGGCTGAGGTTGC	CCCAGTCTGATAGGATGTGTTGG
**RPL13a**	AAAAAGCGGATGGTGGTTC	CTTCCGGTAGTGGATCTTGG
**SOX9**	AGCGAACGCACATCAAGAC	CTGTAGGCGATCTGTTGGGG
**ACAN**	TGCATTCCACGAAGCTAACCTT	GACGCCTCGCCTTCTTGAA
**COL2A1**	GAACCCTGCTCGCACCTG	GACGCAAGTCTCGCCAGTCT
**ALPL**	ATCTTTGGTCTGGCCCCCATG	AGTCCACCATGGAGACATTCTCTC
**RUNX2**	CAACCACAGAACCACAAGTGCG	TGTTTGATGCCATAGTCCCTCC
**IBSP**	GAACCTCGTGGGGACAATTAC	CATCATAGCCATCGTAGCCTT
**SPP1**	TCACCAGTCTGATGAGTCTCACCATTC	TAGCATCAGGGTACTGGATGTCAGGT
**BGLAP**	CACTCCTCGCCCTATTGG	CCCTCCTGCTTGGACACAAAG
**COL10A1**	GTGTTTTACGCTGAACGATACCAA	ACCTGGTTTCCCTACAGCTGATG
**COL1A1**	GAGGGCCAAGACGGAGACATC	CAGATCACGTCATCGCACAAC
**MMP1**	TGCAACTCTGACGTTGATCCCAGA	ACTGCACATGTGTTCTTGAGCTGC
**MMP3**	GTGAGGACACCAGCATGAA	GACCACTGTCCTTTCTCCTAAC
**MMP13**	ACTGAGAGGCTCCGAGAAATG	GAACCCCGCATCTTGGCTT
**ADAMTS4**	CAGACAGCCCTCCATCTAAAC	CCCTTCCCTGGTGCTAAATAA
**ADAMTS5**	GCACTGGCTACTATGTGGTATT	AGCCAGTTCTCACACACTTC

### 2.7 Biochemical assays

For all biochemical assays, spent medium was collected from the cartilage only plate, as well as the syringes from the bioreactor system. Medium was centrifuged at 10000xg for 10 minutes and the supernatant was collected. SensoLyte 520 Generic matrix metalloproteinase (MMP) Activity Kit Fluorimetric (AnaSpec, Fremont, CA) was performed per the manufacturer’s instructions. DuoSet ELISA for total human MMP-1, MMP-3, and MMP-13 (R&D Systems, Minneapolis, MN) were performed per manufacturer’s instructions on spent medium diluted up to 1000X as necessary.

### 2.8 Statistical analysis

Normalized values of all assays were imported into GraphPad Prism 10 (La Jolla, CA) for plotting and analysis. RM Friedman test (paired, nonparametric) with Dunn’s posthoc was performed for all datasets. Due to high standard deviations and donor variability, p-values < 0.2 were reported on the graphs in grey, and p-values < 0.05 were reported in black. Any different statistical test performed were stated in the respective figure caption.

## 3 Results

### 3.1 Inflammatory conditions negatively affect cartilage anabolic markers

**Figure 2:**
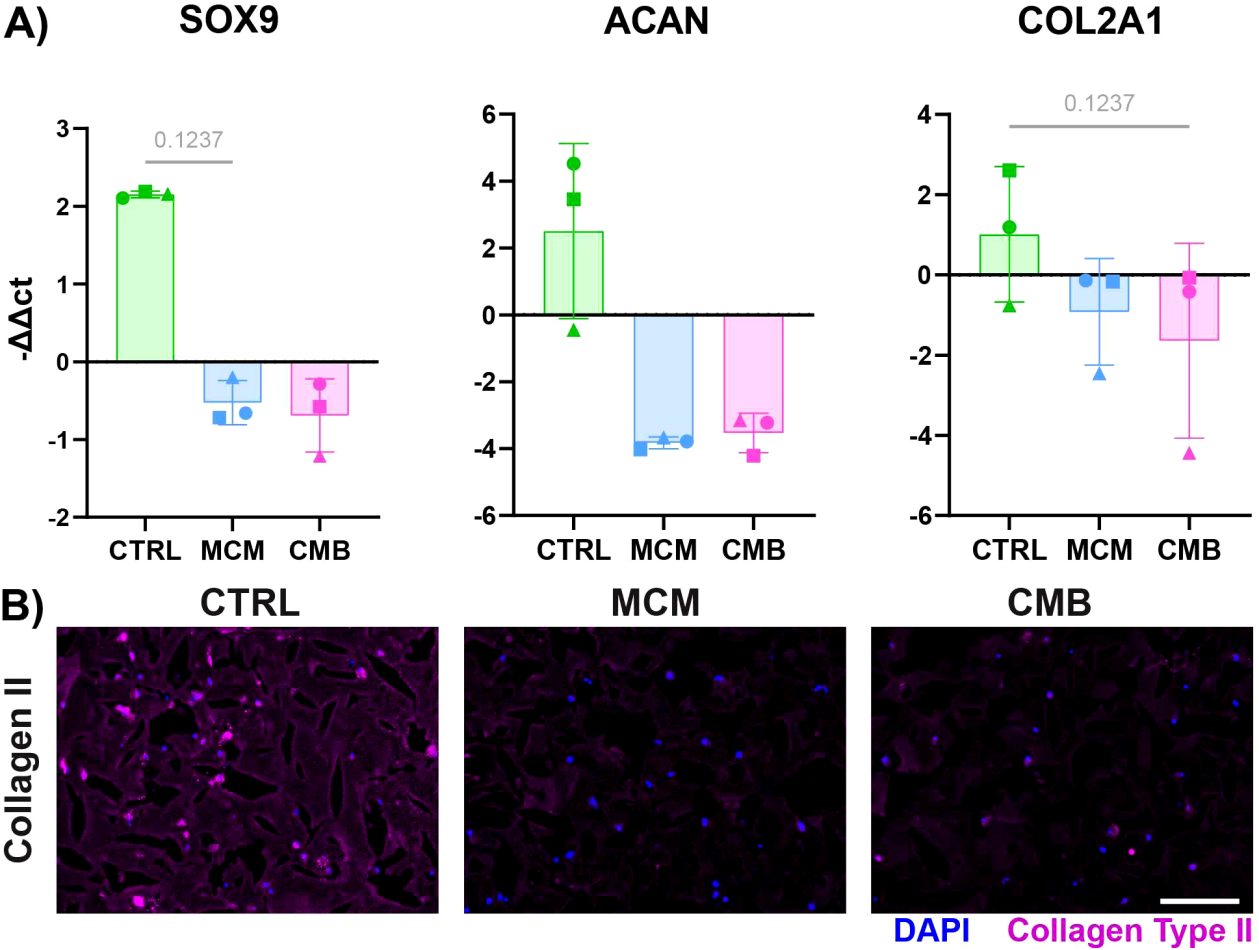
Inflammatory conditions decrease cartilage anabolism in isolated cartilage constructs. **(A)** RT-qPCR data showing a decrease in expression of chondrogenic genes *SOX9*, *ACAN*, *COL2A1* under inflammatory conditions (n=3 pools, 3 donors/pool, normalized to D0 untreated and HKG *RPL13a*, Friedman P values: *SOX9 =* 0.1944, *ACAN* = 0.1944, *COL2A1 =* 0.1944). **(B)** Immunofluorescent staining for collagen type II showing a decrease in signal under inflammatory conditions (Scale = 50μm).

To first establish the baseline effects that different inflammatory conditions have on cartilage, we administered either MCM or CMB to cartilage constructs cultured in a multi-well plate for 7 days. RT-qPCR showed downregulation of chondrogenic genes for both inflammatory conditions compared to the control of chondrogenic medium without any inflammatory agents (**Figure 2A**). Specifically, we looked at SRY-Box Transcription Factor 9 (*SOX9)*, the master regulator of chondrogenesis, *ACAN*, a gene encoding for the proteoglycan aggrecan that is abundant in cartilage, and *COL2A1*, the gene for collagen type II, the primary collagen in cartilage ECM. To corroborate, there was decreased staining for collagen type II in the matrix of the cartilage gel constructs in the inflammatory conditions (**Figure 2B**, **Supplementary Figure 3**), specifically the MCM condition.

### 3.2 Macrophage conditioned media causes strong matrix remodeling in isolated cartilage constructs

**Figure 3:**
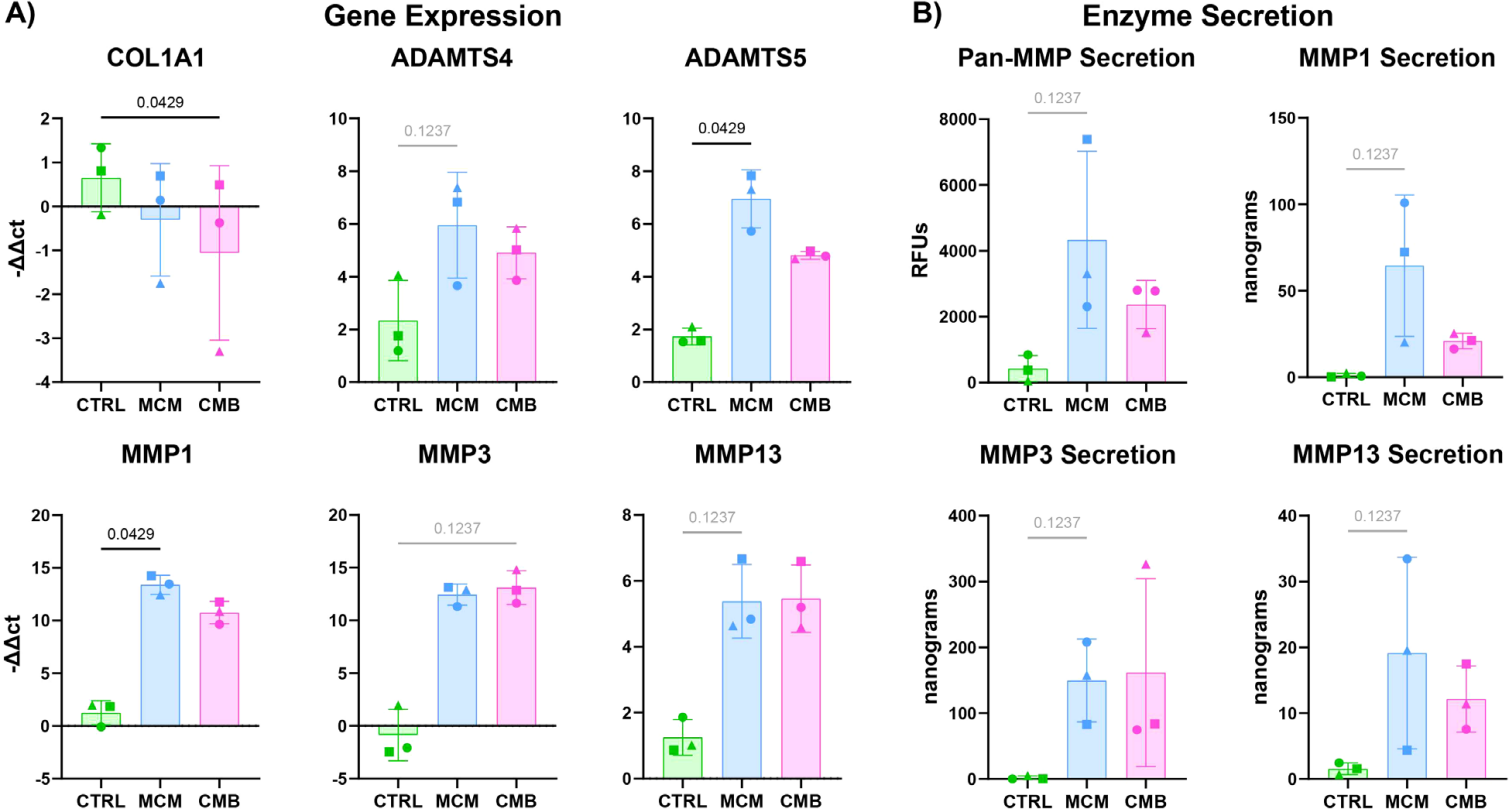
Inflammatory conditions increase catabolic genes in isolated cartilage constructs. **(A)** RT-qPCR data showing an increase in catabolic genes in the *ADAMTS* family and *MMP* family in inflammatory conditions (n=3 pools, 3 donors/pool, normalized to D0 untreated and HKG *RPL13a*, Friedman P values: *COL1A1 =* 0.0278, *ADAMTS4 =* 0.1944, *ADAMTS5 =* 0.0278, *MMP1 =* 0.0278, *MMP3 =* 0.1944, *MMP13 =* 0.1944). **(B)** Pan-MMP assay and MMP1, 3, and 13 ELISA data showing an increase in MMP secretion under inflammatory conditions (n=3 pools, 3 donors/pool, blanked with media and normalized to volume and construct number, Friedman P values: Pan-MMP = 0.1944, MMP1 = 0.1944, MMP3 = 0.1944, MMP13 = 0.1944).

The change in matrix composition and matrix remodeling factors such as MMPs were also measured in the individually cultured cartilage gel constructs in response to inflammatory cues. There was upregulation of *MMP1*, *MMP3*, *MMP13*, *ADAMTS4*, and *ADAMTS5* in the inflammatory conditions compared to control (**Figure 3A**). *MMP1* and *ADAMTS5* were significantly upregulated in the MCM condition over the CMB condition. There was significant downregulation of *COL1A1*, the gene encoding for collagen type I, in the CMB group. To corroborate the gene expression findings, Pan-MMP assay and ELISAs were performed on the collected spent media. Pan-MMP assay showed continuous secretion of MMPs over the 7-day course of inflammatory cues delivery, with the highest MMP secretion detected in the MCM group (**Figure 3B**, **Supplementary Figure 4**). The Pan-MMP assay showed the abundance of the entire MMP family in the supernatant. To determine the specific activity of key MMPs implicated in arthritis, we performed ELISAs for MMP1, MMP3, and MMP13. The results from the ELISAs mirrored this trend for all key MMPs, showing greater secretion in the MCM group compared to the CMB group (**Figure 3B**, **Supplementary Figure 4**). There was little to no secretion of MMPs in the CTRL group as expected.

### 3.3 Inflammatory conditions cause catabolic response in the cartilage component of the cartilage-vascularized bone constructs, but with a different profile than isolated cartilage constructs

**Figure 4:**
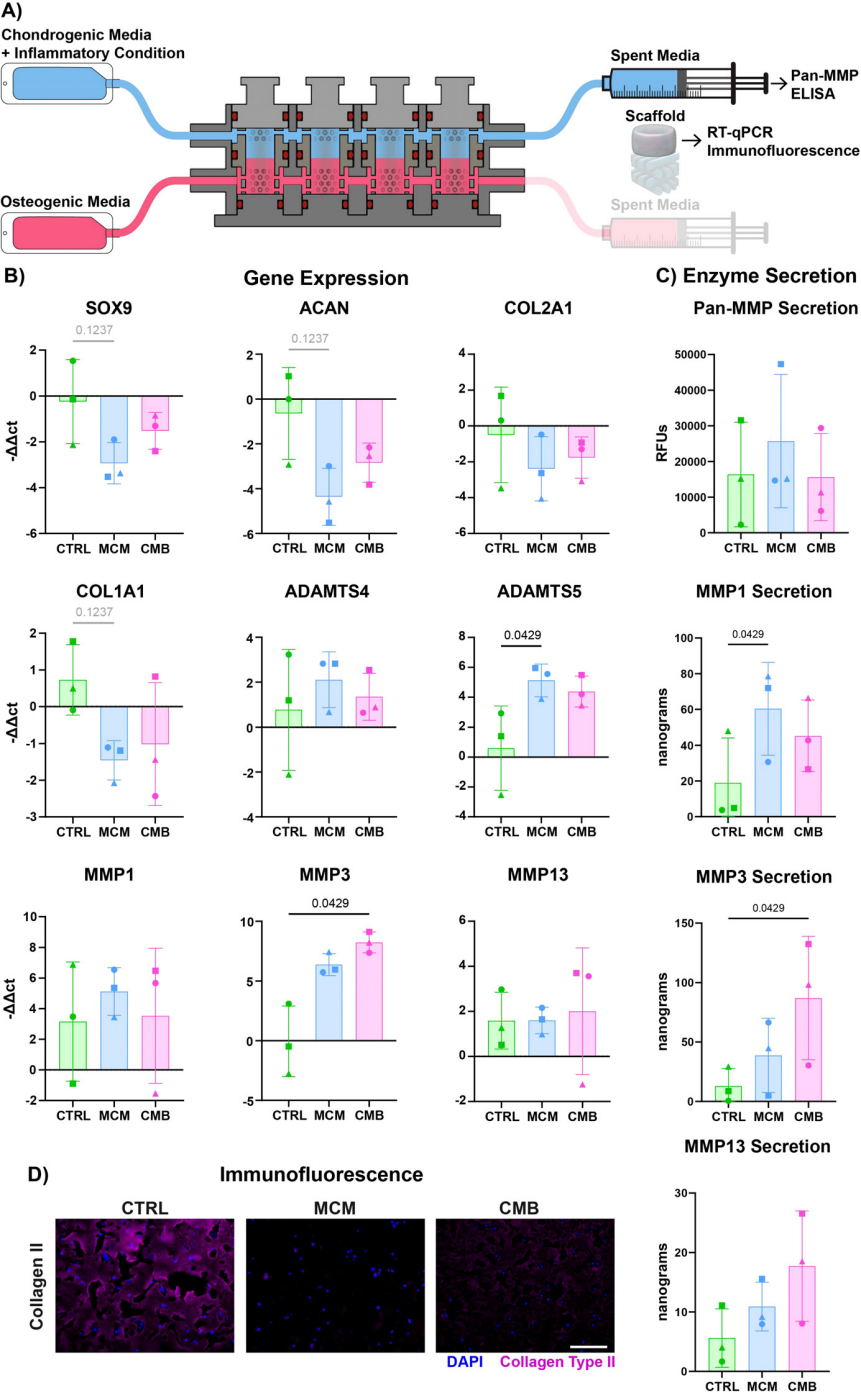
Inflammatory profile differs slightly in coculture cartilage constructs. **(A)** Diagram explaining the sample and media collection from the bioreactor coculture. **(B)** RT-qPCR data showing both a decrease in cartilage anabolic genes and increase in cartilage catabolic genes in inflammatory conditions (n=3 pools, 3 donors/pool, normalized to D0 untreated and HKG *RPL13a*, Friedman P values: *SOX9 =* 0.1944, *ACAN *= 0.1944, *COL2A1 =* 0.3611, *COL1A1 =* 0.1944, *ADAMTS4 =* 0.9444, *ADAMTS5 =* 0.0278, *MMP1 =* 0.9444, *MMP3 =* 0.0278, *MMP13 =* 0.9444). **(C)** Pan-MMP assay and MMP1, 3, and 13 ELISA data showing an increase in MMP secretion under inflammatory conditions (n=3 pools, 3 donors/pool, blanked with media and normalized to volume and construct number, Friedman P values: Pan-MMP = 0.3611, MMP1 = 0.0278, MMP3 = 0.0278, MMP13 = 0.3611). **(D)** Immunofluorescent staining for Collagen Type II showing a decrease in signal under inflammatory conditions (Scale = 50μm).

To determine if the addition of the vascularized-bone construct affected the response of the cartilage analogs to inflammatory cues, complete osteochondral constructs were loaded into the bioreactor and underwent inflammatory stimulation for 7 days (**Supplementary Figure 5**). The inflammatory agents in the MCM and CMB groups were perfused only through the cartilage construct in the upper chamber. The constructs were collected for RT-qPCR and histology, and the spent medium was used for biochemical assays (**Figure 4A**). We saw that chondrogenesis is more robust in the bioreactor coculture versus the isolated cartilage constructs in a plate (**Supplementary Figure 6**). RT-qPCR revealed a similar trend to that in the isolated cartilage constructs, with a downregulation of chondrogenic genes *SOX9*, *ACAN*, and *COL2A1* for both MCM and CMB conditions (**Figure 4B**). We similarly saw downregulation of *COL1A1* in MCM group, and significant upregulation of *ADAMTS5* in the MCM, and *MMP3* in the CMB. Notably, expression of MMP1, MMP13, and ADAMTS4 was ameliorated in the inflammatory conditions in coculture. Pan-MMP assay showed higher overall MMP secretion for all groups compared to isolated cartilage constructs, but with lesser differences (**Figure 4C**). Interestingly, while the MCM elicited the greatest MMP 1, 3, and 13 secretions in the isolated cartilage constructs, it appeared that the CMB group drove the greatest MMP 1 and 13 secretion in the presence of the vascularized-bone construct, whereas MMP1 was significantly upregulated in the MCM group. The reduction of collagen II immunofluorescent staining in the inflammatory groups mirrored that of the isolated cartilage construct (**Figure 4D**, **Supplementary Figure 7**). We also compared the *COL2A1/COL1A1* ratio of isolated versus cocultured cartilage constructs, but saw no clear trend (**Supplementary Figure 8A**). Additionally, we compared the expression of endochondral ossification genes bone morphogenetic protein 2 (*BMP2*), vascular endothelial growth factor (*VEGF*), Indian hedgehog homolog (*IHH*), and collagen type 10 (*COL10A1*) (62–64) in both the isolated cartilage construct and the coculture cartilage constructs. We saw significant upregulation of *BMP2* in isolated cartilage constructs under MCM inflammation, but this decreased in cocultured constructs.

### 3.4 Inflamed cartilage causes both anabolic and catabolic response in the vascularized-bone matrix

**Figure 5:**
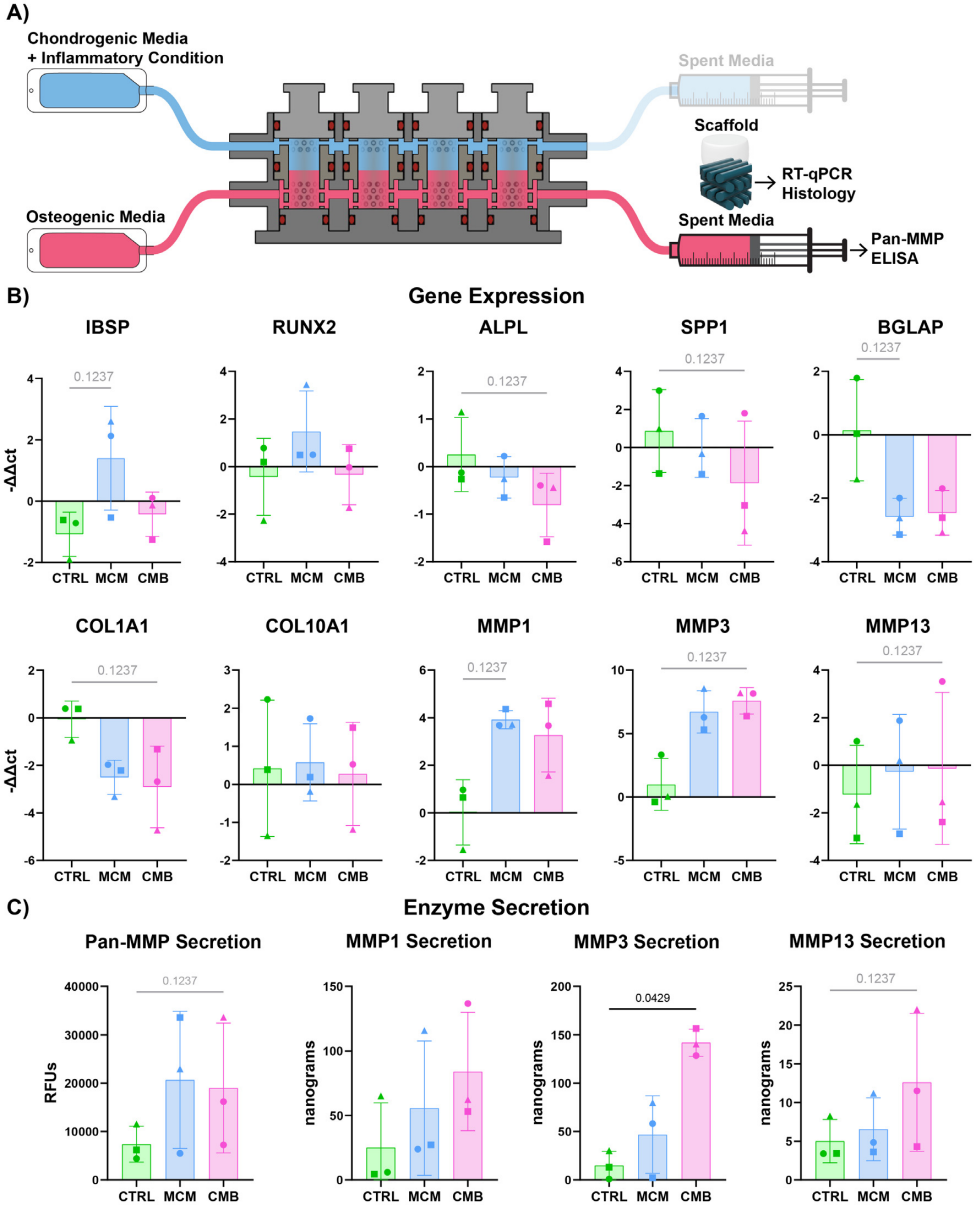
Inflammatory profile of vascularized-bone construct shows evidence of cartilage-bone crosstalk. **(A)** Diagram explaining the sample and media collection from the bioreactor coculture. **(B)** RT-qPCR data showing both a decrease in most bone anabolic genes and increase in bone catabolic genes in inflammatory conditions (n=3 pools, 3 donors/pool, normalized to D0 untreated and HKG *RPL13a*, Friedman P values: *IBSP* = 0.1944, *RUNX2 = * 0.9444, *ALPL* = 0.1944, *SPP1 = * 0.1944, *BGLAP* = 0.1944, *COL1A1 = * 0.1944, *COL10A1* > 0.9999, *MMP1 = * 0.1944, *MMP3 = * 0.1944, *MMP13 = * 0.1944). **(C)** Pan-MMP assay and MMP1, 3, and 13 ELISA data showing an increase in MMP secretion under inflammatory conditions (n=3 pools, 3 donors/pool, blanked with media and normalized to volume and construct number, Friedman P values: Pan-MMP = 0.1944, MMP1 = 0.5278, MMP3 = 0.0278, MMP13 = 0.1944).

To determine if there was evidence of crosstalk induced inflammation in the vascularized-bone region, we repeated RT-qPCR and enzyme assays on the vascularized-bone constructs and supernatant (**Figure 5A**). We saw downregulation of bone anabolic genes alkaline phosphatase biomineralization associated (*ALPL*), secreted phosphoprotein 1 (*SPP1*), and bone gamma-carboxyglutamate protein (*BGLAP*) in inflammatory conditions (**Figure 5B**). We saw upregulation of osteogenic genes runt-related transcription factor 2 (*RUNX2*) and integrin binding sialoprotein (*IBSP*) in the MCM condition only. There was a decrease of *COL1A1* and an increase of *MMP3* and *MMP13* in the CMB condition, and an increase of MMP1 in CMB and more so MCM. We saw highest Pan-MMP and MMP13 expression in CMB condition, and significantly higher MMP3 expression in the CMB condition (**Figure 5C**).

## 4 Discussion

In this study, we developed an *in vitro* micro-physiological system to model cartilage-bone crosstalk in arthritic environments. To study the inflammatory response of the cartilage analog, we exposed isolated cartilage constructs in a plate to our pro-inflammatory macrophage conditioned medium and cytokine cocktail and compared to the control. Then, we exposed the cartilage region of an osteochondral construct to the same inflammatory conditions and compared the response of the coculture cartilage to the isolated cartilage to determine how the coculture affected the inflammatory response profile. Lastly, to probe crosstalk in our system, we analyzed which evidence of an inflammatory response was shown in the osseus region when the cartilage region was exposed to inflammatory conditions.

We first exposed isolated cartilage constructs to pro-inflammatory stressors to tease out the different responses between CMB and the MCM. *In vivo* during arthritis, cartilage degrades via aggrecanases and metalloproteinases, losing critical ECM components such as collagen type II and GAGs (17). These phenotypes and their associated gene expression are what we looked for when analyzing the response to inflammatory agents of our cartilage constructs. From RT-qPCR and biochemical assays we observed that both inflammatory conditions resulted in cartilage degradation, although the MCM and CMB conditions presented different inflammatory profiles. There was an increase in MMPs and ADAMTSs, and a decrease in Collagen II and aggrecan. The cartilage construct response to MCM seemed to have greater variability, suggesting a potential stronger response. Furthermore, immunofluorescent staining showed that MCM drove a more substantive decrease in collagen type II signal than the CMB condition. This suggests that the MCM comprises inflammatory agents beyond the inflammatory cytokines in the CMB that may contribute to arthritis initiation and progression. However, isolated cartilage constructs are a somewhat limited *in vitro* model when the whole articular joint is involved.

We then repeated the test of the same pro-inflammatory conditions within our biphasic bioreactor combining the cartilage constructs with a vascularized osseous construct in a vascularized osteochondral model. We assessed the impact of crosstalk with the adjacent vascularized-bone construct on the cartilage layers when exposed to a pro-inflammatory environment. We put particular care in including HUVECs within the bone scaffold, so that the construct was both vascularized and mineralized. In fact, previous work from our group when developing the vascularized-bone construct (41) showed that the addition of a vascular component to the bone analog supported more robust osteogenesis via paracrine signaling (65–67). In fact, *in vivo*, vasculature plays a critical role in bone development, maintenance, and regeneration (13), besides supplying oxygen and nutrients from blood. Recently, the Zelzar lab has even proposed that vasculature supports bone growth during development by acting as a collagen I template guiding osteoblast growth and mineralization (68). On the other hand, the MSCs included in our constructs acted as pericytes and stabilized the forming vessels (69), and the MSC-derived osteoblasts in the construct secreted VEGF to support endothelial cell growth and differentiation (70–72). This interconnected relationship between angiogenesis and osteogenesis is preserved in *in vitro* engineered bone constructs (73). Thus, we included vascularization in our model to ensure robust osteogenesis and a more realistic *in vitro* analog.

Bone and cartilage work together *in vivo* during both homeostasis and disease, so we expected the addition of the vascularized-bone construct to alter the response of the cartilage construct to inflammation. BMPs secreted by osteoblasts and osteocytes (74) have been shown to protect cartilage from inflammation (75). In contrast, Hopkins et al. showed that damaged subchondral bone tissue inhibited cartilage GAG production *in vitro* (76). The vascularization in the bone also affects cartilage. Endothelial cells that support osteogenesis may also activate the receptor activator of nuclear factor-κB ligand (RANKL) pathway that regulates cartilage resorption (77) and secrete MMPs that remodel both bone and cartilage. Nagao et al. showed that targeting VEGF, a critical factor in osteogenesis and angiogenesis coupling, attenuates osteoarthritis progression in cartilage (78). Furthermore, vasculature has been shown to activate the phosphatidylinositol 3-kinase and protein kinase B (PI3K/Akt) and BMP pathways (42, 67, 79, 80), which help chondrogenic differentiation both *in vivo* during development, and *in vitro*. Whether the vascularized-bone construct ameliorated or worsened the cartilage construct response to inflammation, we anticipated the crosstalk between the two regions to change in inflammation. Interestingly, we saw the secretion of MMPs was greater in the CMB condition compared to the MCM, which differed from the isolated cartilage results. This suggests that crosstalk between the bone and cartilage does in fact contribute to cartilage inflammatory response, and that factors secreted by the vascularized-bone construct regulate the cartilage construct’s inflammatory response profile. This reinforces the concept that an isolated cartilage construct is hardly sufficient to recapitulate the dynamics occurring in arthritis, and that a robust *in vitro* model should include the critical articular tissues of vascularized-bone.

This also raises interest in the difference between MCM and CMB, and how they elicit different responses in the cartilage construct and the vascularized-bone construct. When deciding what cytokines to include in the combined cytokine cocktail, it was critical to focus on cytokines most implicated in arthritis (54, 81–83). Among these, IL-1*β*, IL-6, and TNF-*α* play a substantive role in both rheumatoid arthritis and osteoarthritis (82, 83). Inflammation in the synovium is the driver of cytokine production in joint disease (81), with synovial fibroblasts producing IL-6 (23) during arthritis progression, whereas macrophages produce IL-1β and TNF-α (11, 54). In the design of the CMB, we started with the concentrations of these three cytokines in the MCM and multiplied tenfold. Arthritis development *in vivo* occurs over decades with physiological levels of these cytokines, so we then chose to consistently use supraphysiological levels to ensure a response in our *in vitro* model in experimentally reasonable time frames. Supraphysiological levels of cytokine are often necessary to elicit a response in 3D cultures due to cytokine interaction with and diffusion through the gel (84, 85), and the protective nature of 3D culture (86, 87). The tenfold multiplication placed the cytokine concentrations in the ranges found most commonly in literature for 3D studies (55, 88, 89) and kept it proportional to the levels in our MCM to maintain consistency. We chose to use a combination of the cytokines instead of testing them individually to represent the *in vivo* environment more closely. Interestingly, there is evidence of crosstalk between the IL-6 and IL-1β pathways (90, 91), and IL-6 has been shown to regulate the IL-1β and TNF-α signaling (92). The cytokines work synergistically to drive disease and should then be studied together. While the cytokines chosen in this study have been shown to have the strongest effect on inflammation, there are of course far more than 3 cytokines that contribute to arthritis. The modularity and convenience of our system means that many different cytokines could be tested. Moving forward, one might consider teasing out the role of other cytokines hypothesized to have a role in arthritis such as IL-10, IL-12, and IL-8, as well as chemokines such as CCL20 and CCL2 (23, 81, 83).

This very same reasoning is also why we chose to test pro-inflammatory macrophage conditioned medium as an inflammatory agent. *In vivo*, synovial macrophages release many pro-inflammatory signals that are major drivers of arthritis. While there has been significant research into the secretome of synovial macrophages, the specifics of its composition and effects are still being decoded (18, 93–95). Nevertheless, we used macrophage conditioned medium to test if it may better represent in our model the *in vivo* arthritic conditions, driving a response more analogous to *in vivo* inflammation. Although, macrophages polarized *in vitro* differ from macrophages *in vivo* (96), we hypothesized that the macrophage conditioned medium would still be sufficient to drive a response in cartilage. We saw that the MCM induces inflammation similar to—and in some cases greater than—that of the CMB condition. For simplicity, using a combination of cytokines successfully induced an inflammatory response *in vitro*, however, the MCM more severely depleted extracellular collagen II protein expression. Since loss of critical ECM components is a hallmark of cartilage degradation in arthritis, this suggests that the MCM can derive an inflammatory response that is more functional and clinically relevant. As for why the MCM may drive a greater inflammatory response, pro-inflammatory macrophages secrete an abundance of proteins (97) that account for many more signaling pathways than those activated by TNF-α, IL-6, and IL-1β. We then hypothesized that the other cytokines secreted by the macrophages, such as IL-12 or IL-23, or chemokines, such as CXC chemokine ligand 1 (CXCL1) and CXCL2 (98, 99), contributed to further response by the construct. Another hypothesis is that pro-inflammatory macrophages secreted nitric oxide (NO) (100), and NO has long been associated with degradation of articular cartilage in arthritis (101). Ideally, the MCM would be a more accurate representation of the synovial fluid in the joint space than the cytokine cocktail, but further secretory analysis of the MCM, and especially the synovial fluid of arthritic patients would be necessary to support this. At this point, we know that synovial fluid from arthritic patients contain cytokines like IL-1, IL-6 (82), and IL-20 (83), and proteases like MMP1 and MMP3 (102). Pro-inflammatory macrophages similarly express MM1 and MMP3 (103), and we confirmed here that the conditioned media contains cytokines IL-1β, IL-6, and TNF-α. There are clearly similarities between conditioned media from pro-inflammatory macrophages and arthritic synovial fluid, but more research is required to precisely determine the full extent of this similarity.

We then looked at the effect of the inflammatory agents on the vascularized-bone analog to determine if the cartilage construct’s inflammation would initiate a response in the vascularized-bone construct. It is important to emphasize that the vascularized-bone construct was never directly perfused with inflammatory agents, and the effect of inflammatory agents onto the vascularized-bone region would be mediated by the cartilage construct. *In vivo*, during arthritic disruption, inflammation dysregulates the RANKL signaling and produces inflammatory cytokines that cause osteoclast-mediated bone erosion (20). Furthermore, IL-1*β* blocks mineralization and disrupts osteoblasts (20), and results in strong secretion of MMPs by both osteocytes and endothelial cells in arthritic environments (10). Therefore, we expected to see a decrease in bone mineralization agents and an increase in MMP secretion in our vascularized-bone region in response to inflammation. Notably, we did see an increase in MMP1, 3, and 13 gene expression and protein secretion in the vascularized-bone construct in both CMB and MCM inflammatory conditions. We also saw a decrease in osteogenic genes in the CMB condition. Unexpectedly, we observed a slight increase in *RUNX2* and *IBSP* in the MCM group, implying there may be something in the MCM that promotes expression of those genes. One potential explanation is that inflamed and resorbed bone release BMPs (104), and BMPs can trigger both *RUNX2* and subsequent *IBSP* upregulation (105). Since the vascularized-bone construct presented an inflammatory response profile even when MCM or CMB were delivered only to the cartilage construct, this suggests that the inflamed cartilage construct signals to the bone construct and propagates the inflammation and disease. Thus, we confirmed that our *in vitro* model allows for crosstalk between the cartilage analog and the bone analog, recapitulating some aspects of the crosstalk that occurs *in vivo* during disease.

As an even closer model to *in vivo* circumstances, one might consider using explants in the biphasic bioreactor, and several research groups have created successful *ex vivo* models using tissue explants (106, 107). However, animal explants suffer from the same limitation as the use of animal cells in terms of lower biological similarity to humans. Human explants would be ideal, but they are more challenging to source in sufficient number for screening and can more easily be obtained by joint replacement surgeries, hence, from joints that present varying and inhomogeneous degrees of tissue degeneration. Therefore, creating a biomimetic osteochondral construct with human cell sources is a good option to create a biologically similar human analog while allowing for significant replicates appropriate for the future screening of therapeutics.

Overall, the results of our study showed that our micro-physiological system is a valid model of crosstalk between bone and cartilage in an arthritic environment. We showed that macrophage conditioned media generated an inflammatory response profile in the cartilage constructs that may be more clinically relevant compared to a cytokine cocktail, suggesting that the latter may not capture the full extent of the inflammatory profile of the synovium in arthritis. Furthermore, we showed that the combination with a vascularized-bone analog in an osteochondral construct modulated the cartilage analog’s response to inflammatory agents, pointing to crosstalk between the cartilaginous and vascularized-osseous components. Notably, the main benefit of our model is the ability to deliver agents to one compartment within the biphasic bioreactor to study the effect of crosstalk between the two tissues. This unique feature could be leveraged to explore the effects of specific molecules in modeling or modulating arthritis, or to test the efficacy of therapeutics in a good throughput system prior to preclinical animal studies.

## Data Availability

The original contributions presented in the study are included in the article/**Supplementary Material**. Further inquiries can be directed to the corresponding author.
