# Supplementary material for: Developing an in vitro osteochondral micro-physiological system for modeling cartilage-bone crosstalk in arthritis
Source: Front Immunol. 2025 May 26;16:1495613. doi: 10.3389/fimmu.2025.1495613 (PMC12146386; doi:10.3389/fimmu.2025.1495613)
Supplement: Supplementary file 1 [file DataSheet1.docx]

Supplementary Figures:


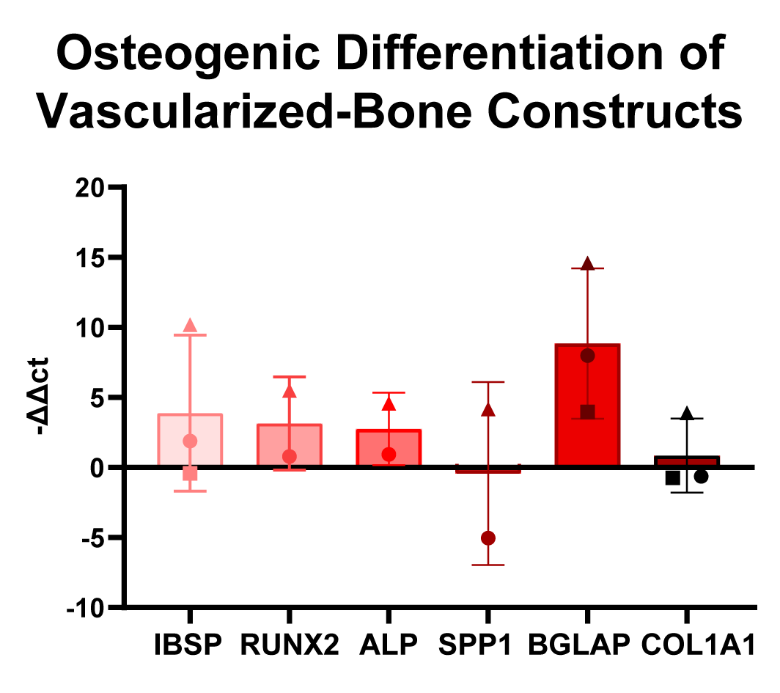


**Supplementary Figure 1: Confirmation of osteogenesis on bone scaffold prior to HUVEC addition.** RT-qPCR data showing an increase in bone anabolic genes after 14 days. (One sample Wilcoxon, n=3 pools, 3 donors/pool, normalized to D0 differentiation and HKG *RPL13a*, sample loss occurred for pool 2 *RUNX2* and *ALPL*).


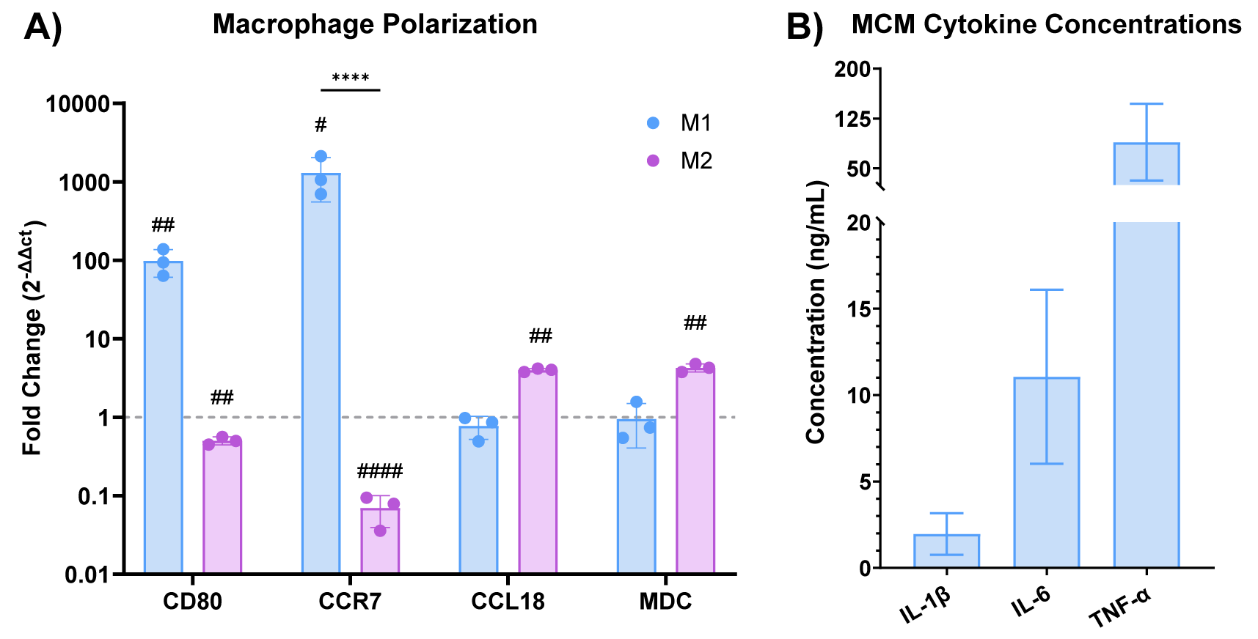


**Supplementary Figure 2: Macrophage polarization and cytokine secretion.** A) RT-qPCR data showing expression of canonical M1 and M2 markers in M1 and M2 polarized macrophages (2 way ANOVA and Unpaired T-test, n=3 replicates, normalized to HKG 18S and time-matched M0 macrophages, * represents M1 compared to M2 expression, ****<0.0001, # represents expression vs time-matched M0, #<0.05, ##< 0.01, ####<0.0001). B) ELISA data showing concentrations of IL-1β, IL-6, and TNF-α in conditioned media collected from M1 macrophages polarized with IFN-γ and LPS.


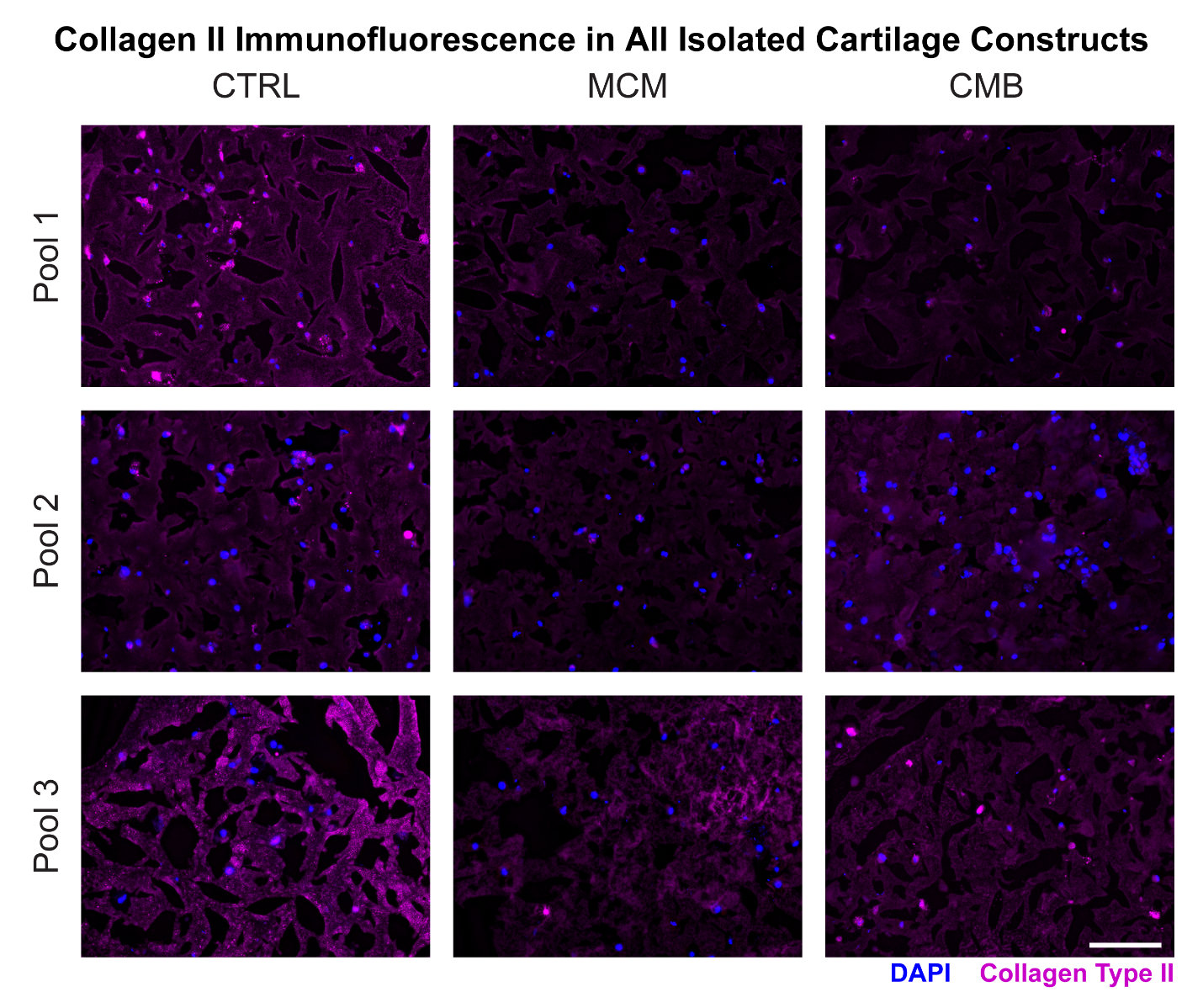
**Supplementary Figure 3: Immunofluorescent staining for Collagen Type II in all conditions of all pools.** Immunofluorescent staining for Collagen Type II (magenta) and DAPI (blue) for all isolated cartilage construct samples (Scale = 50μm).


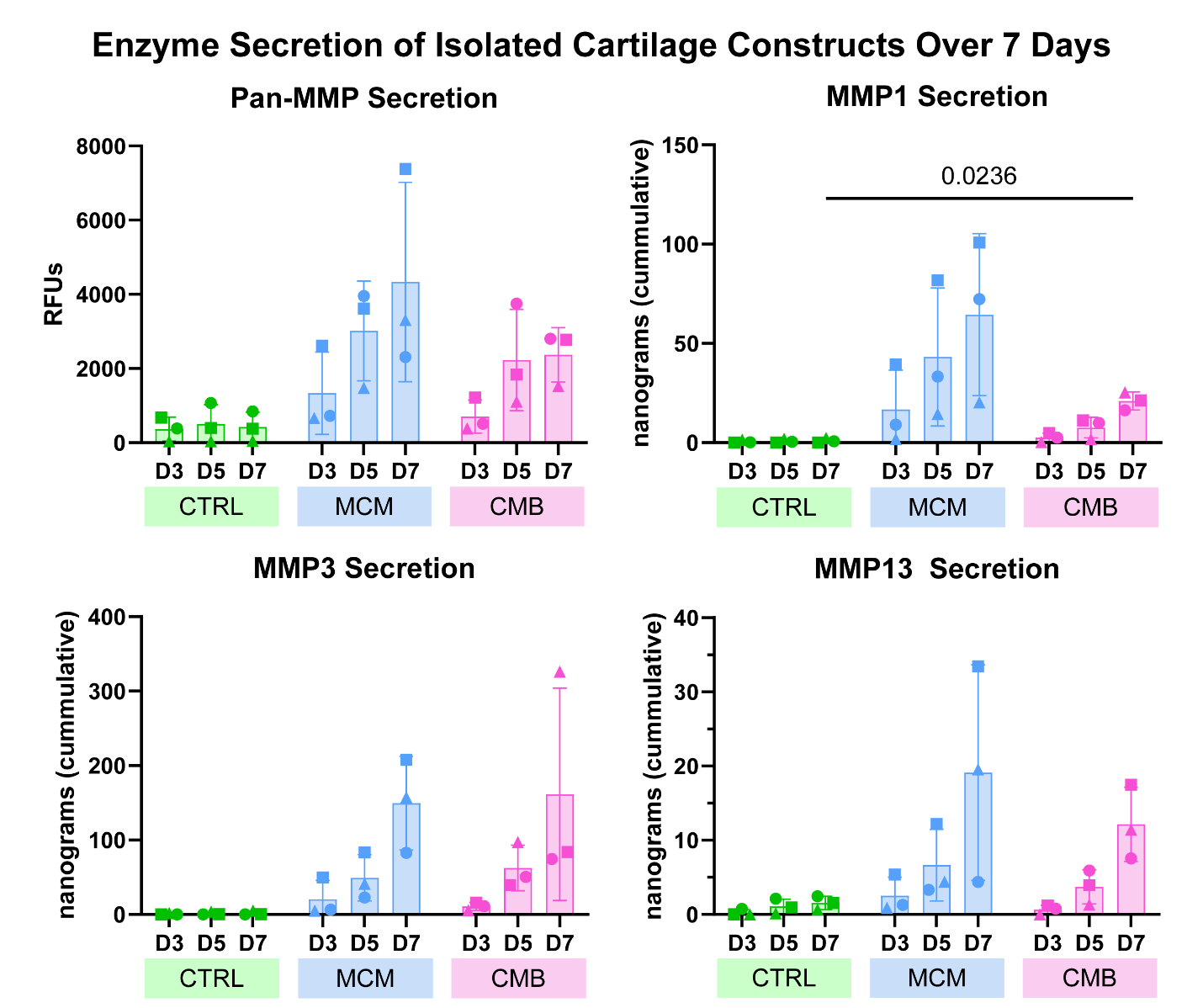
**Supplementary Figure 4: Pan-MMP assay and ELISAs for isolated cartilage constructs over 7 days.** Media supernatants were collected day 3, 5, and 7 for isolated cartilage constructs, and Pan-MMP assay and ELISAs were performed on all samples (2 way ANOVA, n=3 pools, 3 donors/pool, blanked with media and normalized to volume and construct number).


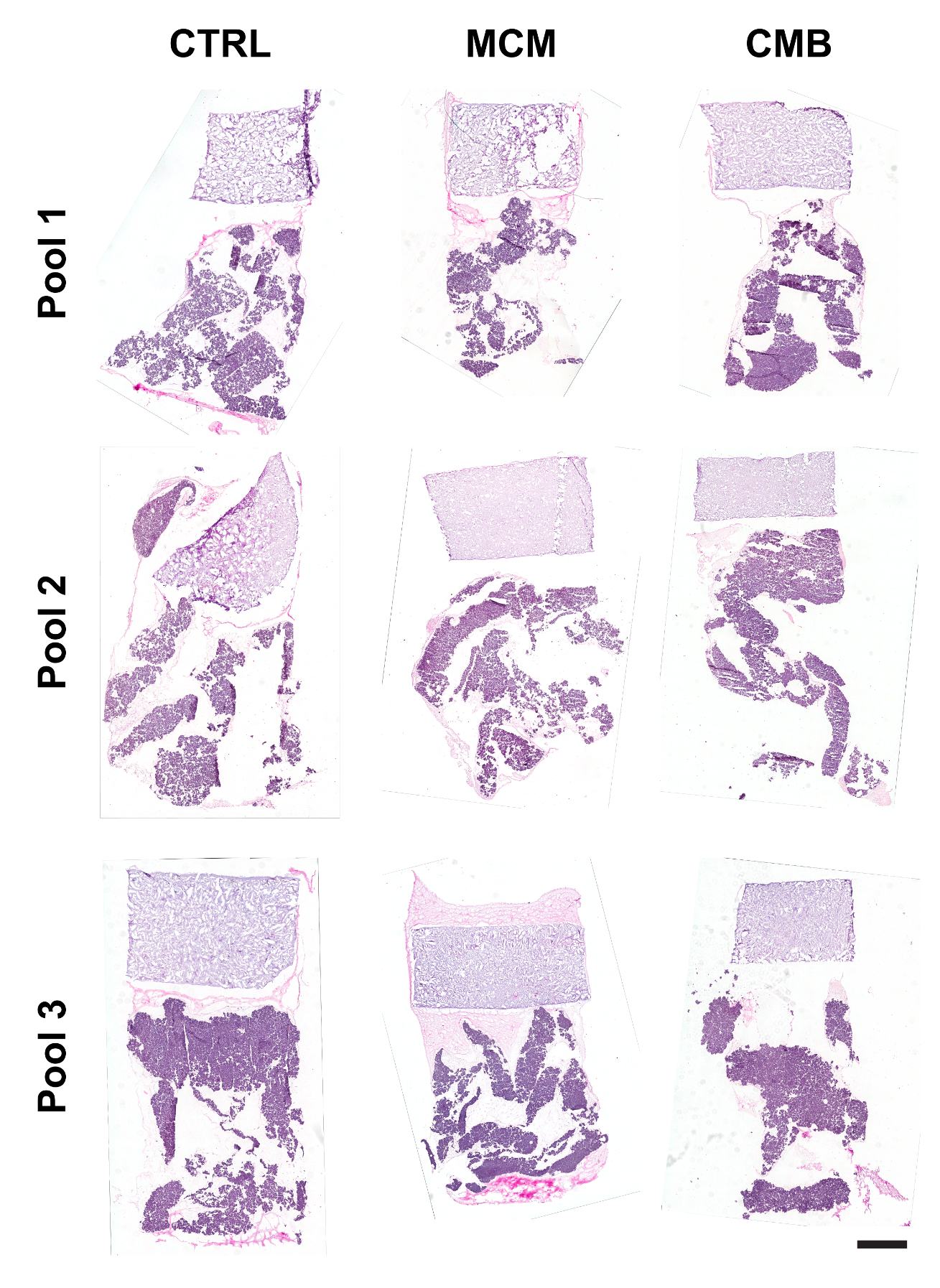
**Supplementary Figure 5: Histological staining of complete osteochondral construct in all conditions of all pools.** Hematoxylin & Eosin staining of complete osteochondral construct after treatment by inflammatory conditions showing the cartilage construct and the vascularized-bone construct (Scale = 1mm).


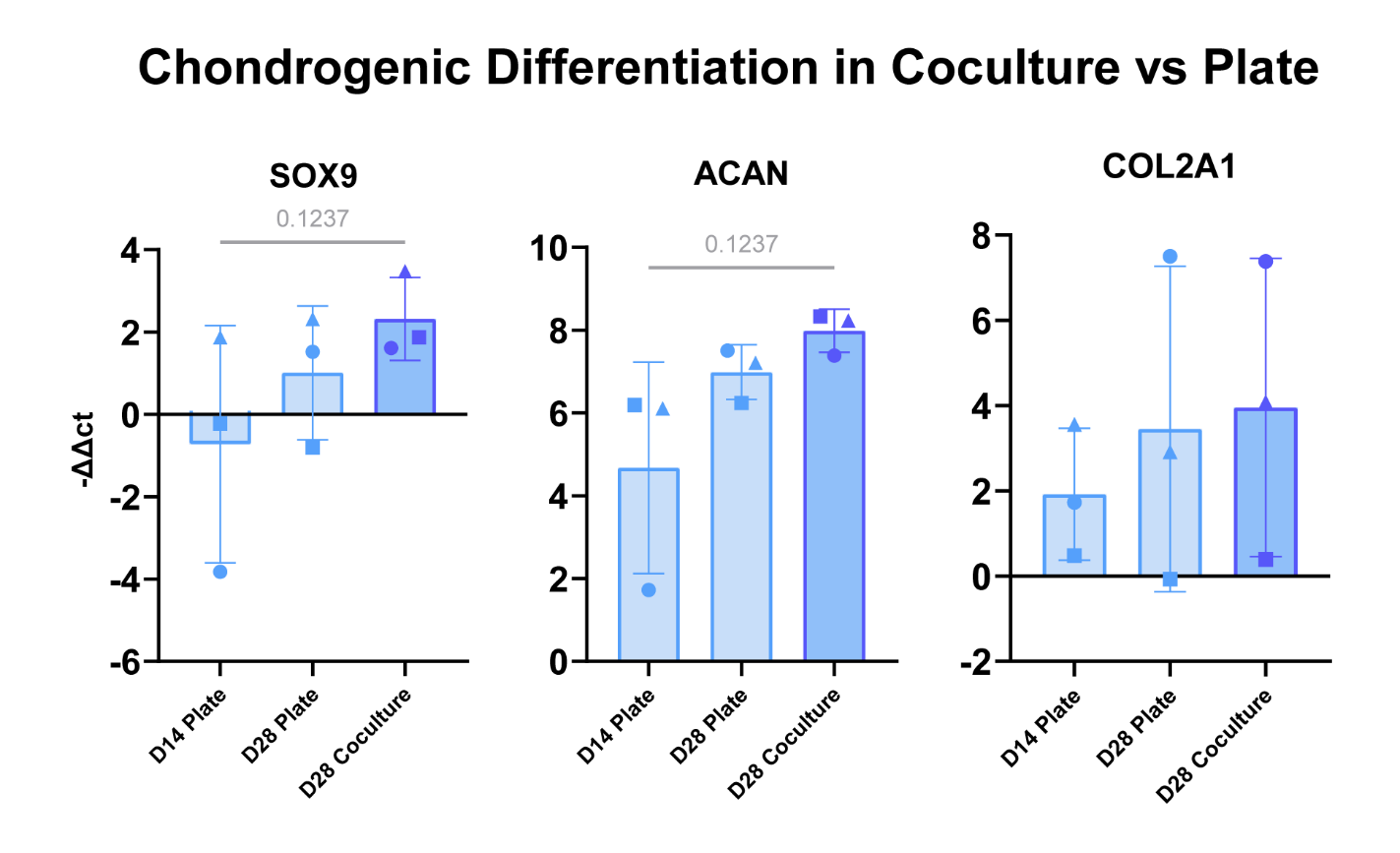
**Supplementary Figure 6: Chondrogenic differentiation in coculture versus a plate.** RT-qPCR data showing increase in chondrogenic anabolic genes at D28 of differentiation in coculture versus D14 plate and D28 plate (n=3 pools, 3 donors/pool, normalized to D0 differentiation and HKG *RPL13a*, Friedman P values: *SOX9*=0.1944, *ACAN*=0.1944, *COL2A1*=0.9444).


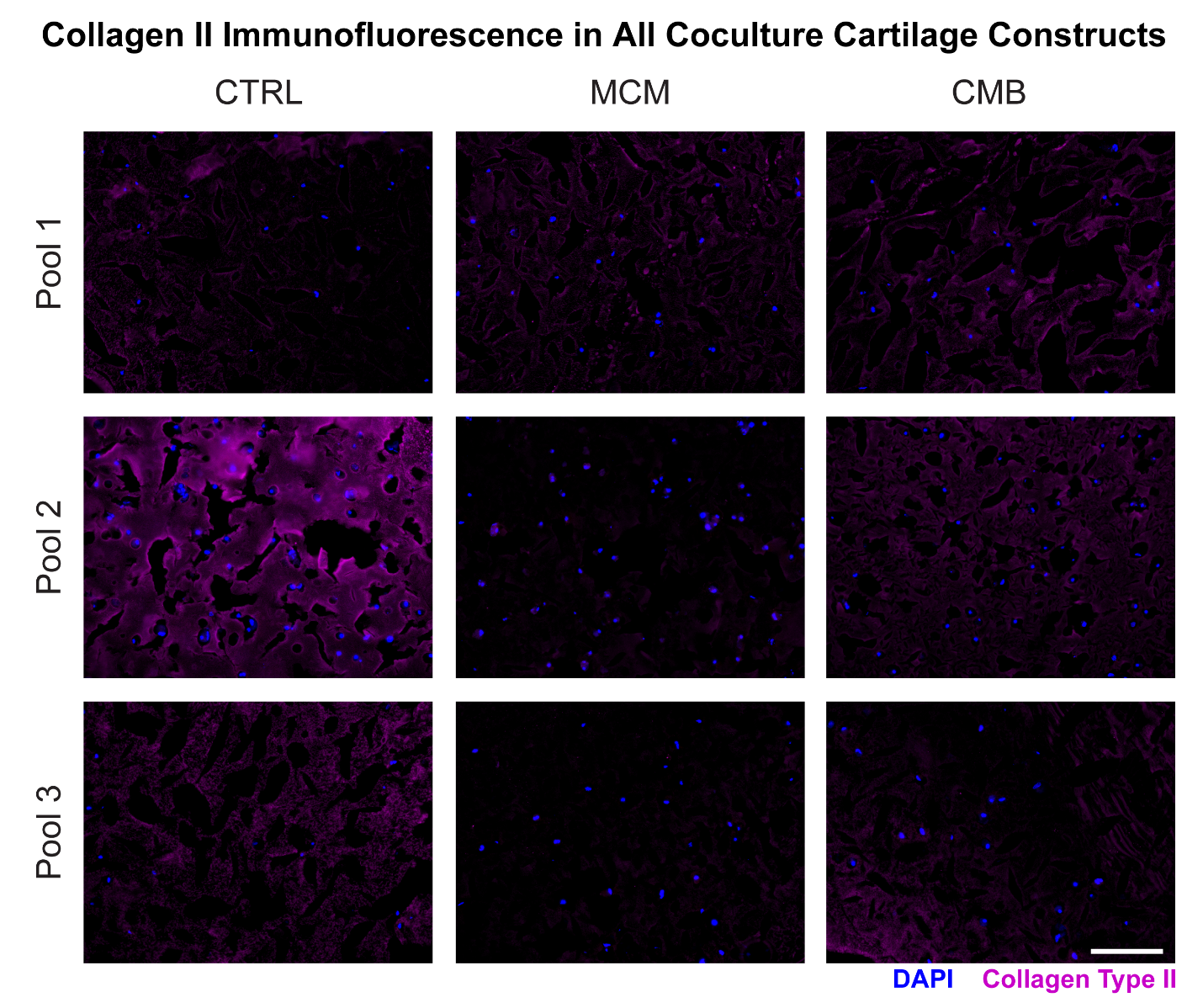
**Supplementary Figure 7: Immunofluorescent staining for Collagen Type II in all conditions of all pools.** Immunofluorescent staining for Collagen Type II (magenta) and DAPI (blue) for all coculture cartilage samples (Scale = 50μm).


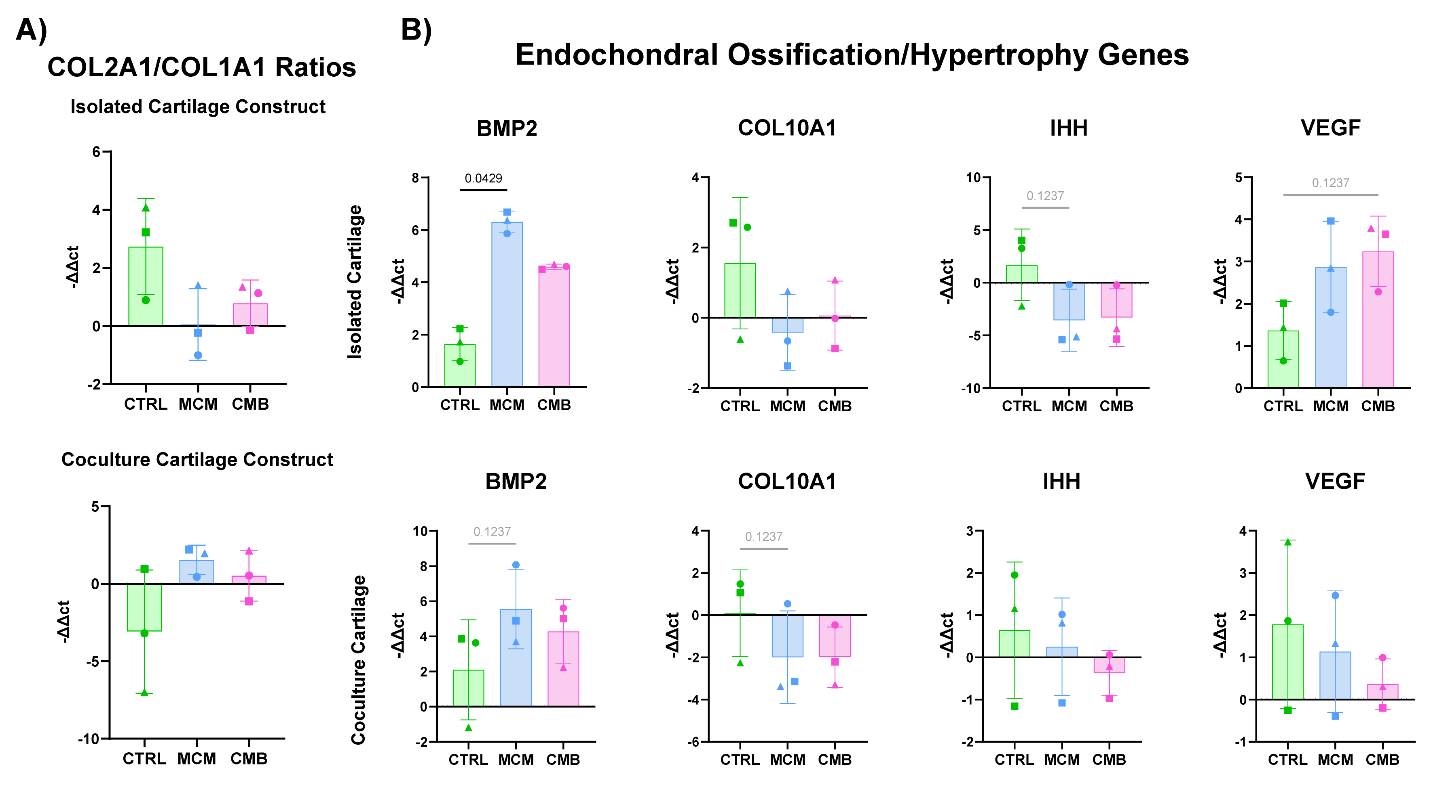
**Supplementary Figure 8: COL2/COL1 ratios and endochondral ossification genes in isolated vs. cocultured cartilage constructs.** A) RT-qPCR showing the *COL2A1*/*COL1A1* ratios in isolated cartilage construct versus coculture cartilage construct (n=3 pools, 3 donors/pool, normalized to D0 treatment and HKG RPL13a, Friedman P Value: Isolated=0.3611, Cocultured=0.5278). B) RT-qPCR showing expression of endochondral ossification or hypertrophy genes in isolated cartilage construct versus coculture cartilage construct (n=3 pools, 3 donors/pool, normalized to D0 treatment and HKG *RPL13a*, Friedman P values right to left and top to bottom: 0.0278, 0.5278, 0.1944, 0.1944, 0.1944, 0.1944, 0.9444, 0.9444).
